# Establishing Statistical Equivalence of Data from Different Sampling Approaches for Assessment of Bacterial Phenotypic Antimicrobial Resistance

**DOI:** 10.1128/AEM.02724-17

**Published:** 2018-04-16

**Authors:** Heman Shakeri, Victoriya Volkova, Xuesong Wen, Andrea Deters, Charley Cull, James Drouillard, Christian Müller, Behnaz Moradijamei, Majid Jaberi-Douraki

**Affiliations:** a Institute of Computational Comparative Medicine, Kansas State University, Manhattan, Kansas, USA; b Department of Anatomy and Physiology, Kansas State University, Manhattan, Kansas, USA; c Department of Diagnostic Medicine/Pathobiology, Kansas State University, Manhattan, Kansas, USA; d Center for Outcomes Research and Epidemiology, Kansas State University, Manhattan, Kansas, USA; e Veterinary and Biomedical Research Center, Inc., Manhattan, Kansas, USA; f Department of Animal Sciences and Industry, Kansas State University, Manhattan, Kansas, USA; g Department of Statistics, Kansas State University, Manhattan, Kansas, USA; h Department of Mathematics, Kansas State University, Manhattan, Kansas, USA; Rutgers, The State University of New Jersey

**Keywords:** Escherichia coli, NARMS, Salmonella, assessment of antimicrobial resistance, bacterial antimicrobial resistance, cattle, fecal sampling, sample processing, sample types, statistical equivalence

## Abstract

To assess phenotypic bacterial antimicrobial resistance (AMR) in different strata (e.g., host populations, environmental areas, manure, or sewage effluents) for epidemiological purposes, isolates of target bacteria can be obtained from a stratum using various sample types. Also, different sample processing methods can be applied. The MIC of each target antimicrobial drug for each isolate is measured. Statistical equivalence testing of the MIC data for the isolates allows evaluation of whether different sample types or sample processing methods yield equivalent estimates of the bacterial antimicrobial susceptibility in the stratum. We demonstrate this approach on the antimicrobial susceptibility estimates for (i) nontyphoidal Salmonella spp. from ground or trimmed meat versus cecal content samples of cattle in processing plants in 2013-2014 and (ii) nontyphoidal Salmonella spp. from urine, fecal, and blood human samples in 2015 (U.S. National Antimicrobial Resistance Monitoring System data). We found that the sample types for cattle yielded nonequivalent susceptibility estimates for several antimicrobial drug classes and thus may gauge distinct subpopulations of salmonellae. The quinolone and fluoroquinolone susceptibility estimates for nontyphoidal salmonellae from human blood are nonequivalent to those from urine or feces, conjecturally due to the fluoroquinolone (ciprofloxacin) use to treat infections caused by nontyphoidal salmonellae. We also demonstrate statistical equivalence testing for comparing sample processing methods for fecal samples (culturing one versus multiple aliquots per sample) to assess AMR in fecal Escherichia coli. These methods yield equivalent results, except for tetracyclines. Importantly, statistical equivalence testing provides the MIC difference at which the data from two sample types or sample processing methods differ statistically. Data users (e.g., microbiologists and epidemiologists) may then interpret practical relevance of the difference.

**IMPORTANCE** Bacterial antimicrobial resistance (AMR) needs to be assessed in different populations or strata for the purposes of surveillance and determination of the efficacy of interventions to halt AMR dissemination. To assess phenotypic antimicrobial susceptibility, isolates of target bacteria can be obtained from a stratum using different sample types or employing different sample processing methods in the laboratory. The MIC of each target antimicrobial drug for each of the isolates is measured, yielding the MIC distribution across the isolates from each sample type or sample processing method. We describe statistical equivalence testing for the MIC data for evaluating whether two sample types or sample processing methods yield equivalent estimates of the bacterial phenotypic antimicrobial susceptibility in the stratum. This includes estimating the MIC difference at which the data from the two approaches differ statistically. Data users (e.g., microbiologists, epidemiologists, and public health professionals) can then interpret whether that present difference is practically relevant.

## INTRODUCTION

Informative assessment of bacterial antimicrobial resistance (AMR) within and among strata is the basic block in any investigation of AMR epidemiology or control approaches (1, 2). Such assessments are critical for identifying influential factors and mitigation strategies for AMR (1, 2). Examples of strata are animal or human populations, food products, environmental areas, manure effluents from food animal farms, and human sewage effluents. To assess AMR of a target bacterial species in a stratum, isolates of the bacteria are obtained from the sampling units (e.g., animal hosts or environmental area segments) in the stratum. Each isolate's phenotypic susceptibility to each target antimicrobial drug is measured as the drug's MIC inhibiting visible overnight growth of the isolate culture (3). The data for all the obtained isolates provide the distribution of the tested antimicrobial's MIC as an estimate for the target bacteria in the stratum. Descriptive statistics (e.g., elemental features of the data such as means, percentiles, or ranges) have been used extensively for the MIC distributions due to the ease of interpretation (the statistics used had been reviewed in detail by Wagner et al. [4]). Such distributions, however, could be subjected to statistical analyses to identify patterns and dynamics of the bacterial antimicrobial susceptibility in the stratum, compare sampling approaches or microbiological sample processing methods for the susceptibility assessments, or contrast the susceptibilities between strata. Analyzing the MIC distributions bears numerous challenges because the distributions tend to have complex shapes (e.g., do not follow the probability distributions commonly assumed for parametric statistical tests) and are inherently censored (i.e., all the isolates with MIC less than or equal to the smallest drug concentration tested are in one category in the beginning of the distribution, and all the isolates with MIC greater than the largest drug concentration tested are in one category in the end of the distribution) (4–7). Thus far, the analytical approaches have included comparing the histograms of relative frequency of the isolates with the specific MICs of the antimicrobial (7) and the cumulative frequency of the isolates over the increasing MICs of the antimicrobial (8, 9) in a stratum over time and between strata. The cumulative frequency distributions have been also used for comparing the antimicrobial susceptibility estimates between the isolate sets from different sampling approaches in a stratum (10). Survival analysis has been adapted to compare the probabilities of isolates with the specific MICs of the antimicrobial (the time to event is replaced by the concentration achieving bacterial growth inhibition, MIC) in a stratum over time and between strata defined by experimental factors (6, 11). Linear regression on the log_2_(MIC) has been used to compare the susceptibility to the antimicrobial in a stratum over time and between strata in the probabilistic framework (12) and to compare the MIC measurements obtained for the same strain set by different microbiological laboratories in the Bayesian framework (5). It has been suggested that a power analysis should be included for the statistical tests of tendencies in the MIC/log_2_(MIC) distributions to support interpretation of the results (12).

Different sampling approaches can be used to assess AMR in a target bacterial species in a stratum. For example, different sample types can be collected, from which the bacteria are then isolated. In other situations, once the samples have been collected, those can be subjected to different sample processing methods for bacterial isolation. For example, an aliquot of the sample can be plated on a bacteriological agar and a different number of the bacterial colonies tested for susceptibility to antimicrobials, or multiple aliquots of the sample can be plated and the bacterial colonies from each aliquot tested. The same analytical need arises in both of these scenarios: sampling a stratum by different sample types and applying different sample processing methods to the samples of one type. The need is to determine whether the sampling or the sample processing approaches yield similar estimates of phenotypic antimicrobial susceptibility in the target bacteria in the stratum. This question can be formulated as to whether the approaches yield equivalent estimates of the antimicrobial's MIC distribution for the bacteria in the stratum. This can be addressed by statistical equivalence testing (13, 14). This technique also provides a flexibility for the data users to interpret whether the existing differences between the bacterial susceptibility estimates for the stratum between the sampling or sample processing approaches are practically relevant (as shown below). The objective of this study was to demonstrate the utility of the statistical equivalence testing as a method to compare the bacterial antimicrobial susceptibility estimates for a stratum between sampling approaches (e.g., different sample types or sampling schemes) or sample processing methods.

## RESULTS

### Interpretation of statistical equivalence testing for MIC data from different sampling or sample processing approaches.

**FIG 1:**
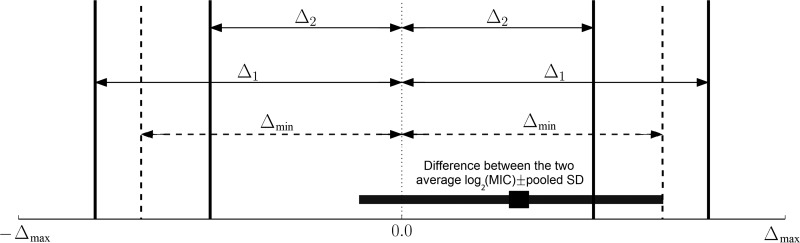
Schematic representation of testing statistical equivalence of the bacterial antimicrobial susceptibility estimates from two sampling or sample processing approaches used in a stratum. If a practically relevant difference between the average log_2_(MIC) estimates from the two approaches is equal to or larger than Δ_min_ (e.g., Δ_1_), the hypothesis of statistical nonequivalence of the estimates will be rejected, signaling equivalence of the MIC data from the two approaches. If a practically relevant difference is smaller than Δ_min_ (e.g., Δ_2_), the hypothesis of statistical nonequivalence of the estimates will be accepted. The maximum possible difference between the log_2_(MIC) values from the two approaches is Δ_max_ $(\Delta_{\text{max}}=\max_{\begin{array}{c} MIC_{1}\in Y_{1}\\ \text{MI}{\text{C}}_{1}\in Y_{1} \end{array}}\left\{|\log_{2}({\text{MIC}}_{1})-\log_{2}({\text{MIC}}_{2})|\right\}$ for the isolate sets *Y*_1_ and *Y*_2_).

**FIG 2:**
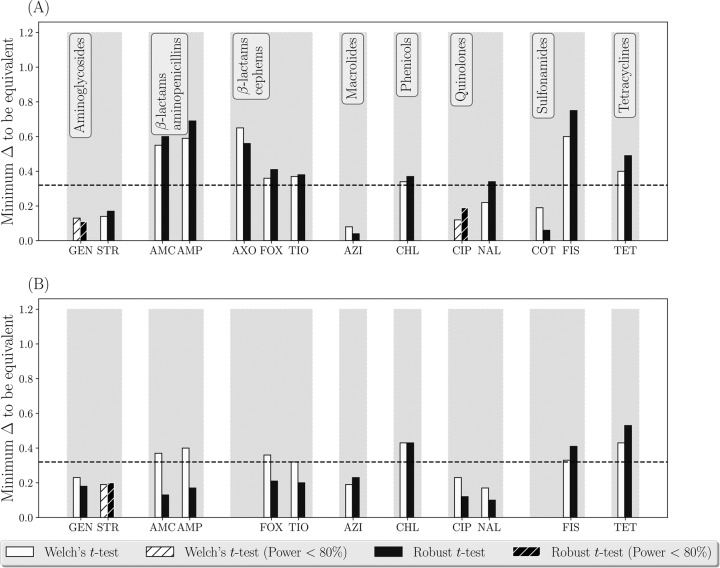
Testing statistical equivalence of the estimates of phenotypic antimicrobial susceptibility of nontyphoidal Salmonella enterica subsp. enterica isolates from the ground- or trimmed-meat samples (*n* = 310 for 2013 and *n* = 344 for 2014) versus cecal content samples (*n* = 435 for 2013 and *n* = 318 for 2014) from cattle in the processing plants in the United States in 2013 (A) and 2014 (B). The data were collected by the NARMS. Aminoglycosides: GEN, gentamicin; STR, streptomycin. β-Lactam aminopenicillins: AMC, amoxicillin-clavulanic acid; AMP, ampicillin. β-Lactam cephems: AXO, ceftriaxone; FOX, cefoxitin; TIO, ceftiofur. Macrolides: AZI, azithromycin. Phenicols: CHL, chloramphenicol. Quinolones: CIP, ciprofloxacin; NAL, nalidixic acid. Sulfonamides: COT, trimethoprim sulfamethoxazole; FIS, sulfisoxazole. Tetracyclines: TET, tetracycline.

**FIG 3:**
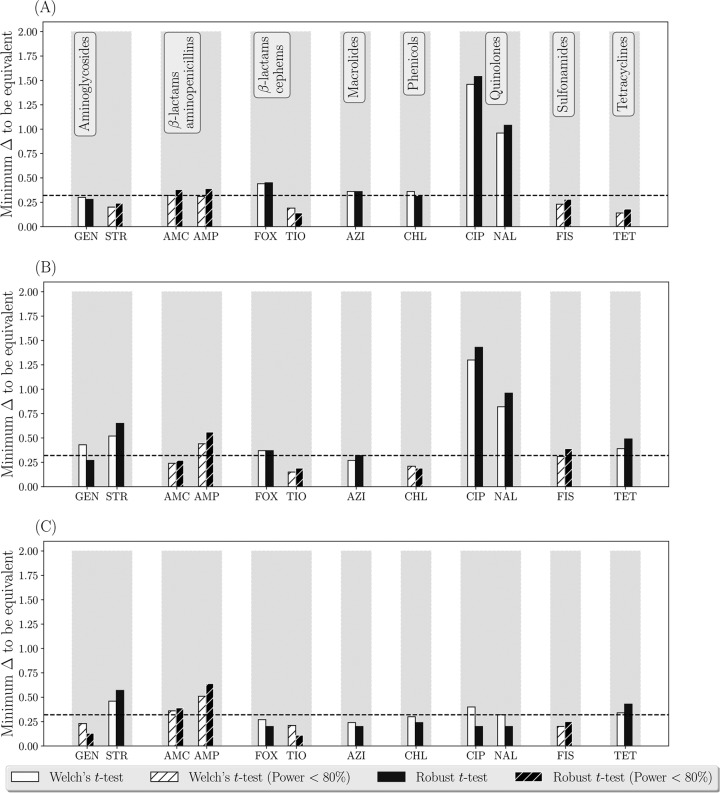
Testing statistical equivalence of the estimates of phenotypic antimicrobial susceptibility of nontyphoidal Salmonella enterica subsp. enterica isolates from urine (*n* = 144), fecal (*n* = 1,495), and blood (*n* = 181) samples of humans in the United States in 2015. (A) Urin*e* versus blood isolates; (B) fecal versus blood isolates; (C) urine versus fecal isolates. The data were collected by the NARMS. Aminoglycosides: GEN, gentamicin; STR, streptomycin. β-Lactam aminopenicillins: AMC, amoxicillin-clavulanic acid; AMP, ampicillin. β-Lactam cephems: FOX, cefoxitin; TIO, ceftiofur. Macrolides: AZI, azithromycin. Phenicols: CHL, chloramphenicol. Quinolones: CIP, ciprofloxacin; NAL, nalidixic acid. Sulfonamides: FIS, sulfisoxazole. Tetracycline: TET, tetracycline.

**FIG 4:**
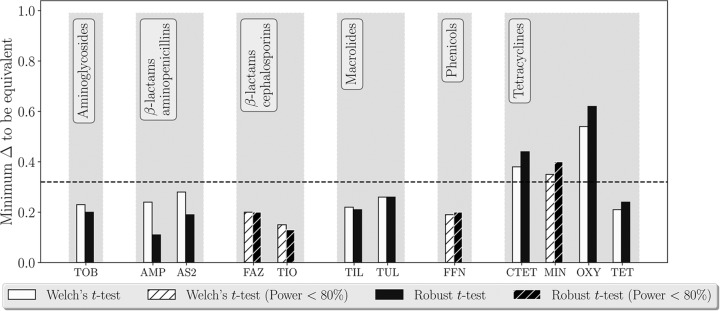
Testing statistical equivalence of the estimates of phenotypic antimicrobial susceptibility of E. coli in cattle fecal pads (*n* = 32). The sample processing approaches compared were testing susceptibility of 4 E. coli isolates obtained from one aliquot of the pad versus testing susceptibility of 4 E. coli isolates each obtained from a different aliquot of the pad, with the aliquots collected from locations spread along the longest axis of the pad. Aminoglycosides: TOB, tobramycin. β-Lactam aminopenicillins: AMP, ampicillin; AS2, ampicillin-sulbactam (2:1 ratio). β-Lactam cephems: FAZ, cefazolin; TIO, ceftiofur. Macrolides: TIL, tilmicosin; TUL, tulathromycin. Phenicols: FFN, florfenicol. Tetracyclines: CTET, chlortetracycline; MIN, minocycline; OXY, oxytetracycline; TET, tetracycline.

The most commonly used measurement of susceptibility of a bacterial isolate to an antimicrobial is the drug's MIC. When the MIC is measured using the broth microdilution assay based on serial 2-fold dilutions of the drug, the measurement is transformed to log_2_(MIC) for statistical analyses (12, 15). The measurements for all the target bacterial species' isolates obtained via a given sampling or sample processing approach from the target stratum yield the antimicrobial's MIC distribution for the species in the stratum. Such distributions from two sampling or sample processing approaches can be compared, and the minimum difference between the average log_2_(MIC) estimates from the two approaches at which the estimates are still statistically equivalent can be determined. We designate that difference Δ_min_. This threshold difference value can be found by performing the statistical equivalence testing on the log_2_(MIC) data from the two approaches starting from a large value of the difference Δ ≫ 1 ≥ Δ_min_ and then reducing it until finding Δ→Δ_min_ below which the hypothesis of a statistically significant difference between the average log_2_(MIC) cannot be rejected. This leads to the estimate of Δ_min_ obtained from the confidence interval (CI) of the difference between the average log_2_(MIC) estimates from the two sampling or sample processing approaches (see Materials and Methods for details). The estimate of Δ_min_ can be interpreted by data users as illustrated in Fig. 1. If a practically relevant difference Δ_1_ is outside Δ_min_, i.e., Δ_1_ ≥ Δ_min_, the two approaches yield statistically equivalent data. In contrast, if a practically relevant difference Δ_2_ is inside Δ_min_, i.e., Δ_2_ < Δ_min_, the two approaches yield statistically nonequivalent data. Thus, data users could apply their perspectives of which difference between the average log_2_(MIC) estimates from the two sampling or sample processing approaches is practically relevant and compare that to the existing statistically significant difference, Δ_min_. Summarizing the results as illustrated in Fig. 2 to 4 enables evaluating the differences in the average log_2_(MIC) estimates between the two sampling or sample processing approaches for individual antimicrobials tested (within and between the drug classes).

Here we provide a suggestion on how Δ_min_ could be interpreted systematically. When bioequivalence of two drug preparations is investigated based on a biological drug response variable for which logarithmic transformations are appropriate, the preparations are considered equivalent if the difference Δ in the variable values is such that 2^Δ^ is ≤1.25 (16). This corresponds to ≤0.32 on the log_2_(MIC) scale. If none of the two sampling or sample processing approaches compared is a reference for the bacterial antimicrobial susceptibility assessment, data users can consider the absolute value of the difference between the average log_2_(MIC) estimates. They could interpret that the two approaches yield nonequivalent estimates of the average log_2_(MIC) if Δ_min_ is >0.32. Such values of Δ_min_ suggest that the estimates differ beyond the biological variation expected if the two approaches were gathering the isolates from the same subpopulation of the target bacteria in the sampled stratum. Note that the statistical determination of Δ_min_ accounts for variability in the data from the two approaches (see Materials and Methods for details).

### Case study 1: ground- or trimmed-meat versus cecal content samples from cattle in processing plants for assessing antimicrobial susceptibility of nontyphoidal Salmonella enterica subsp. enterica in cattle.

Monitoring of AMR in the U.S. food chain is conducted by the National Antimicrobial Resistance Monitoring System (NARMS) (17). In cattle processing plants, both samples of ground or trimmed meat and of cecal contents of cattle carcasses were collected in 2013-2014 (17, 18). Nontyphoidal Salmonella enterica subsp. enterica isolates of diverse serovars were obtained from both these sample types (17, 18). Phenotypic susceptibility of the isolates to antimicrobials representing major antimicrobial drug classes was tested (Table 1) (17, 18) (the data can be found here: https://www.fda.gov/AnimalVeterinary/SafetyHealth/AntimicrobialResistance/NationalAntimicrobialResistanceMonitoringSystem/ucm416741.htm). As the first case study, we investigated statistical equivalence of the average log_2_(MIC) estimates of each tested antimicrobial (Table 1) for S. enterica yielded by the ground- or trimmed-meat samples (*n* = 310 for 2013 and *n* = 344 for 2014) versus cecal content samples (*n* = 435 for 2013 and *n* = 318 for 2014). In both 2013 and 2014, the equivalence testing was used to determine for each antimicrobial Δ_min_, i.e., the difference between the average log_2_(MIC) from the two sampling approaches at which the approaches still yielded statistically equivalent data. The distributions of the log_2_(MIC) for all the tested antimicrobials in each 2013 and 2014 did not follow a normal distribution (Wilk-Shapiro test, *P* value < 0.05 for each of the two distributions from the sampling approaches). Because of this, the robust *t* test was used for the equivalence testing (see Materials and Methods for details). The values of Δ_min_ tended to be larger for phenicols, sulfonamides, and tetracyclines in both 2013 and 2014 (Fig. 2A and B). Relatively large Δ_min_ values were also estimated for β-lactams, both aminopenicillins and cephems, in 2013 (Fig. 2A). The statistical nonequivalence of the log_2_(MIC) data for these drug classes highlighted that the cecal content sampling gauged S. enterica subpopulations that differ in their phenotypic AMR from S. enterica subpopulations gauged via the ground- or trimmed-meat sampling in the cattle processing plants. This interpretation was based on considering the data for each antimicrobial from the two sampling approaches nonequivalent if Δ_min_ was >0.32.

**TABLE 1:** Antimicrobial drug susceptibilities tested for nontyphoidal Salmonella enterica subsp. enterica isolates from ground- or trimmed-meat samples and from cecal content samples collected from cattle in processing plants in the United States in 2013 and 2014^*a*^

Antimicrobial drug class	Subclass	Combinatory formulation	Drug
Aminoglycosides	-Micins	No	Gentamicin
	-Mycins	No	Kanamycin (tested in 2013 only)
		No	Streptomycin
β-Lactams	Aminopenicillins with β-lactamase inhibitors	Yes	Amoxicillin with clavulanic acid
	Aminopenicillins	No	Ampicillin
	Cephems	No	Ceftriaxone
		No	Cefoxitine
		No	Ceftiofur
Macrolides	Azalides	No	Azithromycin
Phenicols	NA	No	Chloramphenicol
Quinolones	Fluoroquinolones	No	Ciprofloxacin
	Quinolones	No	Nalidixic acid
Sulfonamides	NA	Yes	Sulfamethoxazole with trimethoprim
		No	Sulfisoxazole
Tetracyclines	Tetracyclines	No	Tetracycline

a The data were collected by the U.S. National Antimicrobial Resistance Monitoring System. Note that due to a low variability in the data for kanamycin in 2013 and ceftriaxone in 2014, the equivalence testing could not be performed. NA, not applicable.

### Case study 2: antimicrobial susceptibility of nontyphoidal S. enterica subsp. enterica from urine versus fecal versus blood samples of humans.

Monitoring of AMR in enteric pathogens of humans in the United States is also a part of the NARMS activities (17). Nontyphoidal Salmonella enterica subsp. enterica isolates of diverse serovars were obtained from urine, fecal, and blood samples of humans in the United States in 2015 (the analyzed data set is limited to those states that have permitted the U.S. Centers for Disease Control and Prevention to share the data with the public; the data set can be found at https://wwwn.cdc.gov/narmsnow/). Phenotypic susceptibility of the isolates to antimicrobials representing major antimicrobial drug classes was tested (Table 2). As the second case study, we applied the statistical equivalence testing to compare the estimates of antimicrobial susceptibility of nontyphoidal S. enterica isolates from urine (*n* = 144), fecal (*n* = 1,495), and blood (*n* = 181) samples of humans in the United States in 2015. The distributions of the log_2_(MIC) for all the tested antimicrobials for the S. enterica isolates from each urine, fecal, or blood samples did not follow a normal distribution (Wilk-Shapiro test, *P* value < 0.05 for each distribution). Thus, the robust *t* test was used for the equivalence testing. The results demonstrated that susceptibilities of the S. enterica isolates from human urine versus from blood to cephems (which are β-lactams), macrolides, phenicols, and quinolones were statistically nonequivalent (Fig. 3A). Further, susceptibilities of the S. enterica isolates from human feces versus from blood to aminoglycosides, cephems, quinolones, and tetracyclines were nonequivalent (Fig. 3B). Susceptibilities of the S. enterica isolates from human urine versus from feces differed to a lesser extent but still were nonequivalent for aminoglycosides, quinolones, and tetracyclines (Fig. 3C). These interpretations for each antimicrobial in a pairwise comparison of the isolate sources were based on considering the data nonequivalent if Δ_min_ was >0.32. The largest differences were found for the fluoroquinolone ciprofloxacin and the older quinolone nalidixic acid (Fig. 3A and B), to which the isolates from human blood had lower susceptibilities (higher ciprofloxacin and nalidixic acid MICs) than the isolates from either urine or feces. The difference between the average log_2_(MIC) of ciprofloxacin for the isolates from blood versus urine was 1.08 (95% CI: 0.74, 1.42), and for the isolates from blood versus feces it was 1.20 (95% CI: 0.86, 1.54). The difference between the average log_2_(MIC) of nalidixic acid for the isolates from blood versus urine was 0.71 (95% CI: 0.46, 0.95), and for the isolates from blood versus feces it was 0.80 (95% CI: 0.57, 0.96).

**TABLE 2:** Antimicrobial drug susceptibilities tested for nontyphoidal Salmonella enterica subsp. enterica isolates from urine, fecal, and blood samples of humans in the United States in 2015^*a*^

Antimicrobial drug class	Subclass	Combinatory formulation	Drug
Drugs for which variability in the log_2_(MIC) data from urine, fecal, and blood samples was sufficient to perform the statistical equivalence testing			
Aminoglycosides	-Micins	No	Gentamicin
	-Mycins	No	Streptomycin
β-Lactams	Penicillins, including amino-, carboxy-, and ureido-, with β-lactamase inhibitors	Yes	Amoxicillin with clavulanic acid
	Penicillins, including amino-, carboxy-, and ureido-	No	Ampicillin
	Cephems	No	Cefoxitin
		No	Ceftiofur
Macrolides	Azalides	No	Azithromycin
Phenicols	NA	No	Chloramphenicol
Quinolones	Fluoroquinolones	No	Ciprofloxacin
	Quinolones	No	Nalidixic acid
Sulfonamides	NA	No	Sulfisoxazole
Tetracyclines	Tetracyclines	No	Tetracycline
Drugs for which variability in the log_2_(MIC) data from urine, fecal, and blood samples was insufficient to perform the statistical equivalence testing			
β-Lactams	Cephems	No	Ceftriaxone
Sulfonamides	NA	Yes	Sulfamethoxazole with trimethoprim
Drugs for which statistical equivalence testing could not be performed because of a low no. of each urine and blood samples (*n* < 10) tested with the antimicrobials			
β-Lactams	Penicillins, including amino-, carboxy-, and ureido-, with β-lactamase inhibitors	Yes	Piperacillin with tazobactam constant
	Carbapenems	No	Imipenem
	Cephems with β-lactamase inhibitors	Yes	Cefotaxime with clavulanic acid Ceftazidime with clavulanic acid
	Cephems	No	Cefepime
		No	Cefotaxime
		No	Cefquinome
		No	Ceftazidime
	Monobactams	No	Aztreonam

a The data were collected by the U.S. National Antimicrobial Resistance Monitoring System.

### Case study 3: processing a fecal sample for assessing antimicrobial susceptibility of fecal Escherichia coli using multiple bacterial isolates from one aliquot versus one isolate from each of multiple aliquots of the sample.

Assessment of AMR in a target culturable bacterial species in a fecal sample customary involves using a single aliquot from the sample (19, 20). The aliquot is diluted (the dilution is chosen based on the expected bacterial density) and the dilution(s) is plated on a bacteriological agar (19, 20). One or more of the bacterial colonies with typical morphology for the species on the agar are selected, each of the colonies is replated for isolation, and the isolate's phenotypic susceptibility to antimicrobials is tested (19, 20). However, the population of commensal bacteria such as Escherichia coli in feces of an animal or human could consist of genetically diverse subpopulations (21–25). We have chosen to investigate for cattle fecal pads if testing susceptibility of E. coli obtained from a single aliquot of the pad (conventional approach) versus from multiple aliquots taken over the longest axis of the pad yield equivalent estimates of phenotypic antimicrobial susceptibility of fecal E. coli. Fresh fecal pads (*n* = 32) were collected from beef and dairy cattle at research facilities at Kansas State University during May to June 2016. The animals that were sampled had not received antimicrobial drugs in the preceding week, but 1 to 3 weeks prior they had received either antimicrobials or feed supplemented with copper (Cu) or zinc (Zn) (which can coselect AMR in the animal fecal bacteria [11, 26–29]). This animal selection was done to ensure that the cattle fecal E. coli would have detectable but not uniformly high levels of phenotypic AMR, to facilitate statistical analyses of the data. Four aliquots were taken equidistantly along the longest axis of each pad. One of the four aliquots was randomly selected, and four E. coli isolates were obtained from that aliquot. From each of the other three aliquots from the pad, one E. coli isolate was obtained. All the isolates were tested for phenotypic susceptibility to antimicrobials, which were chosen to represent most of the existing antimicrobial drug classes (Table 3). As the third case study, we investigated for each of these antimicrobials the statistical equivalence of the average log_2_(MIC) estimates for fecal E. coli yielded by testing four bacterial isolates from a single aliquot of the fecal pad versus testing one bacterial isolate from each of four aliquots taken from locations spread over the longest axis of the fecal pad. The results demonstrated detectable differences between the two sample processing methods in the estimates of fecal E. coli susceptibility to aminopenicillins (which are β-lactams), aminoglycosides, macrolides, and tetracyclines (Fig. 4). Only for tetracyclines did the two methods yield statistically nonequivalent estimates of fecal E. coli susceptibility. The interpretation for each tested antimicrobial was based on considering the data nonequivalent if Δ_min_ was >0.32. The observed variability in the log_2_(MIC) for antimicrobials of newer classes (Table 3) was insufficient for the testing; the E. coli isolates were predominantly susceptible to these antimicrobials.

**TABLE 3:** Antimicrobial drug susceptibilities tested for Escherichia coli isolates from cattle fecal pads

Antimicrobial drug class	Subclass	Combinatory formulation	Drug
Drugs for which variability in the log_2_(MIC) data from the two sample processing approaches was sufficient to perform the statistical equivalence testing			
Aminoglycosides	-Mycins	No	Tobramycin
β-Lactams	Penicillins, including amino-, carboxy-, and ureido-	No	Ampicillin
	Penicillins, including amino-, carboxy-, and ureido-, with β-lactamase inhibitors	Yes	Ampicillin with sulbactam, 2:1 ratio
	Cephems	No	Cefazolin
		No	Ceftiofur
Macrolides	Macrolides	No	Tilmicosin
	Triamilides	No	Tulathromycin
Phenicols	NA	No	Florfenicol
Tetracyclines	Tetracyclines	No	Chlortetracycline
		No	Minocycline
		No	Oxytetracycline
		No	Tetracycline
Drugs for which variability in the log_2_(MIC) data from the two sample processing approaches was insufficient to perform the statistical equivalence testing			
Aminoglycosides	-Micins	No	Amikacin
		No	Gentamicin
	-Mycins	No	Neomycin
		No	Spectinomycin
β-Lactams	Penicillins, including amino-, carboxy-, and ureido-	No	Penicillin
		No	Piperacillin
	Penicillins, including amino-, carboxy-, and ureido-, with β-lactamase inhibitors	Yes	Piperacillin with tazobactam constant
		Yes	Ticarcillin with clavulanic acid
	Carbapenems	No	Doripenem
		No	Ertapenem
		No	Imipenem
		No	Meropenem
	Monobactams	No	Aztreonam
	Cephems	No	Cefepime
		No	Ceftazidime
		No	Ceftriaxone
Macrolides	Macrolides	No	Tylosin
Nitrofurans	NA	No	Nitrofurantoin
Pleuromutilins	NA	No	Pleuromutilin
		No	Tiamulin
Lincosamides	NA	No	Clindamycin
Quinolones	Quinolones	No	Nalidixic acid
	Fluoroquinolones	No	Ciprofloxacin
		No	Danofloxacin
		No	Enrofloxacin
		No	Levofloxacin
Sulfonamides	NA	No	Sulfadimethoxine
		Yes	Sulfamethoxazole with trimethoprim
Tetracyclines	Glycylcyclines	No	Tigecycline

## DISCUSSION

The three case studies provided above illustrate the utility of statistical equivalence testing (13, 14) for establishing equivalence of data yielded by two sampling or sample-processing approaches for assessing phenotypic antimicrobial susceptibility in a target bacterial species in a stratum. Advantages of the proposed method include the estimation difference in the average log_2_(MIC) estimates, Δ_min_, below which the data from the two approaches are statistically significantly different. This provides data users with flexibility to investigate where practically relevant differences fall relative to the existing differences in the data, as illustrated in Fig. 1. Note that in the proposed method, we determine Δ_min_ between the average log_2_(MIC) estimates from the two sampling or sample processing approaches using a sequential algorithm that retests the statistical nonequivalence hypothesis over a range of the average log_2_(MIC) difference values based on the data from the two approaches. For this, we use statistical tests that accommodate censored data with unequal variances and different shapes of the log_2_(MIC) distributions from the two approaches. This is because the data on an antimicrobial's MIC for a set of bacterial isolates from a stratum are inherently censored (all the isolates with MICs less than or equal to the smallest drug concentration tested are in one category in the beginning and all the isolates with MICs greater than the largest drug concentration tested are in one category in the end of the MIC distribution). The resulting log_2_(MIC) distributions for commonly tested antimicrobial drugs have various shapes that often do not fit to a normal distribution (7). In the proposed method for the equivalence testing, Welch's *t* test (30) relaxes the assumption of equal variances in the two compared log_2_(MIC) distributions and the robust *t* test (31) further improves handling of overdispersion (e.g., presence of long tails in the distributions). We keep the results obtained using both of these tests in Fig. 2 to 4 for illustrative purposes. In further applications, the robust *t* test could be recommended for the log_2_(MIC) distributions that do not follow a normal distribution and demonstrate overdispersion.

In the first case study, we evaluated whether sampling the cecal contents and sampling of ground or trimmed meat in the U.S. cattle processing plants yield equivalent data on antimicrobial susceptibility of nontyphoidal S. enterica subsp. enterica in the processed cattle. The data were collected by the NARMS in 2013-2014 to monitor AMR in the U.S. cattle production chain, and therefore we interpret the results at the same population level. The results showed that S. enterica subpopulations in the cecal content samples may be statistically nonequivalent in their phenotypic antimicrobial susceptibility to S. enterica subpopulations in the ground- or trimmed-meat samples (Fig. 2). Thus, the two sampling approaches yield nonequivalent data for monitoring phenotypic antimicrobial susceptibility in S. enterica in cattle in the processing plants. Possible explanations include decontamination and cross-contamination of cattle carcasses and products within the plant, as well as mixing of different carcass parts for the ground meat (32–34). These processes can reduce the role of the cattle intestinal contents as a source of S. enterica in the meat or ground-meat products.

In the second case study, we evaluated statistical equivalence of the antimicrobial susceptibility estimates for nontyphoidal S. enterica isolates from urine, fecal, and blood samples of humans in the U.S. in 2015. The data were collected by the NARMS to monitor AMR in human enteric pathogens in the United States, and thus again we interpret the results at the same population level. The results demonstrated that susceptibilities of the S. enterica isolates from human blood are nonequivalent to those from urine or feces for several major antimicrobial drug classes, such as aminoglycosides, cephems (β-lactams), macrolides, phenicols, quinolones, and tetracyclines (Fig. 3A and B). Lesser differences were observed between the isolates from urine versus feces (Fig. 3C). The differences of largest magnitude were found for quinolones, with the isolates from human blood being less susceptible than those from urine or feces to the fluoroquinolone ciprofloxacin and the older quinolone nalidixic acid (see Results for more details). This could be due to the common use of fluoroquinolones, e.g., ciprofloxacin, as one of the first-line treatment choices for treating serious infections by nontyphoidal salmonellae in human adults (35–38). Another common treatment choice is cephalosporins (β-lactams), e.g., ceftriaxone (the other choices include combinatory formulations containing β-lactams and β-lactamase inhibitors, aminoglycosides, and, as the last resort, polymyxins and carbapenems [β-lactams]) (35, 38). Considering the data for 2015, susceptibilities to individual cephems of the S. enterica isolates from human blood were less different from (although statistically nonequivalent to) those of the isolates from urine or feces, compared to the differences observed for quinolones (Fig. 3A and B). Notably, across the human nontyphoidal Salmonella isolates, the frequency of those with reduced ciprofloxacin susceptibility has been continuously rising and the frequency of those with reduced ceftriaxone susceptibility has overall increased in the United States since 1996 (38, 39).

In the third case study, we evaluated whether testing four bacterial isolates from a single aliquot of the cattle fecal pad versus testing one bacterial isolate from each of four aliquots taken from locations spread over the longest axis of the pad yield equivalent data on phenotypic antimicrobial susceptibility of fecal E. coli at the population level (*n* = 32 pads were tested). The results showed that the two sample processing methods yield statistically nonequivalent estimates of E. coli susceptibility to tetracyclines, with smaller but detectable differences for aminopenicillins (β-lactams), aminoglycosides, and macrolides (Fig. 4). These antimicrobial drug classes have been used in food animals in the United States for the longest periods (40). Tetracyclines, penicillins, and aminoglycosides were introduced in the 1940s and macrolides in the 1970s (40). Consequently, multiple genes encoding various degrees of susceptibility to these drug classes have been observed in fecal E. coli and S. enterica isolates from farm animals (41–45). The tetracycline resistance gene pool is especially diverse, with several tens of *tet* genes described to date for different animal and human hosts (41, 46). Testing E. coli throughout the fecal pad may capture more of the present diversity in the susceptibility to tetracyclines than testing E. coli at a single location in the pad. Also, the statistical power of the equivalence testing depends not only on the sample size but also on variability in the log_2_(MIC) data. Strongly bimodal (less variable) log_2_(MIC) distributions for newer antimicrobial drug classes, due to the high frequencies of the highly susceptible bacterial isolates, impede the testing (see Tables 1 to 3 for examples).

Diagnostic microbiologists consider one 2-fold dilution of the antimicrobial drug to be an acceptable variation in the MIC measurement for an individual bacterial isolate in the broth microdilution assay (47). If such variation occurs randomly among the isolates in the two sampling or sample processing approaches, it is accounted for in the variance component of a statistical test of the data (for example, see Materials and Methods). Such random variation does not bias comparisons of the data between the approaches. However, if the variation is nonrandom and has a systematic source, it could bias the comparisons. Consider an extreme case of the MIC being skewed by one 2-fold drug dilution for every isolate obtained from one sampling or sample processing approach but not from the other approach, e.g., if the samples from one approach were examined in one laboratory and the samples from the other approach in another laboratory. The data user believes that the antimicrobial's MIC measurements in one laboratory are consistently one 2-fold dilution higher or lower than the MIC measurements in the second laboratory. In this case, there would be a difference Δ ≥ 1 for the antimicrobial between the average log_2_(MIC) estimates from the two laboratories. A statistically significant difference beyond that would be manifested as Δ_min_ > 1.

We have included a suggestion for interpreting Δ_min_ > 0.32 as evidence of statistical nonequivalence of the log_2_(MIC) data for the antimicrobial for the bacterial species in the stratum between the two sampling or sample-processing approaches. This interpretation illustrated in Fig. 2 to 4 is an adaptation of a method for establishing bioequivalence of two drug preparations based on values of a biological drug response variable (16). Other standardized interpretations may be proposed in the future for decision-making on whether the two sampling or sample processing approaches are interchangeable or yield equivalent data on phenotypic antimicrobial susceptibility of the target bacteria (i.e., assess susceptibility in the same bacterial subpopulation) in the stratum.

## MATERIALS AND METHODS

### Statistical equivalence testing. (i) Rationale for testing the statistical equivalence hypothesis.

Let *M*_1_ and *M*_2_ be the sampling or sample processing approaches that yield samples *Y*_1_ and *Y*_2_, respectively, of isolates of the target bacterial species from the stratum. The samples *Y*_1_ and *Y*_2_ represent subpopulations *P*_1_ and *P*_2_ of the species in the stratum. The isolate susceptibility to a target antimicrobial is measured (e.g., in our three case studies the susceptibility was measured using the broth microdilution assay and the obtained MICs were log_2_ transformed for the analysis). The statistics μ_1_ and μ_2_ represent the central tendencies of the susceptibility of the unknown source subpopulations *P*_1_ and *P*_2_. “Conventional” hypothesis testing focuses on rejecting *H*_0_ of no statistically significant difference between the central tendencies *H*_0_: μ_1_ − μ_2_ = 0; *H*_*a*_: μ_1_ − μ_2_ ≠ 0. However, even if *H*_0_ is rejected, this provides no proof in favor of *H_a_*. Importantly, testing the conventional *H*_0_ delivers no information for what μ_1_ − μ_2_ difference signals that the central tendencies of the samples *Y*_1_ and *Y*_2_ are statistically significantly different.

### Equivalence hypothesis.

The equivalence hypothesis testing can provide the sought information on a statistically significant μ_1_ − μ_2_. The null and alternative hypotheses are defined as follows (13, 14):

(1) $$\begin{array}{c} H_{0}:\left|\mu_{1}-\mu_{2}\right|>\Delta \\ H_{a}:\left|\mu_{1}-\mu_{2}\right|\leq \Delta \end{array}$$

The equivalence hypothesis testing in equation 1 indicates that the samples *Y*_1_ and *Y*_2_ obtained by the approaches *M*_1_ and *M*_2_ have equal means up to an acceptable tolerance Δ with a predefined confidence interval 1 − 2α (where α is probability of the type I error). This null hypothesis is rejected if the data provide evidence of the equivalence of the means. Otherwise, the null hypothesis of a statistically significant μ_1_ − μ_2_ difference is accepted.

### (ii) Student’s *t* test of the equivalence hypothesis.

The samples *Y*_1_ and *Y*_2_ are obtained by the sampling or sample processing approaches *M*_1_ and *M*_2_. The sample means *Ȳ*_1_ and *Ȳ*_2_ are employed as the point estimators of μ_1_ and μ_2_, with standard errors *se*_1_ and *se*_2_. Therefore, the difference μ_1_ − μ_2_ can be estimated by *Ȳ*_1_ − *Ȳ*_2_ with a standard error $\sqrt{{se}_{1}^{2}+{se}_{2}^{2}}$, which is equal to

(2) $$\sqrt{\frac{\sigma_{1}^{2}}{n_{1}}+\frac{\sigma_{2}^{2}}{n_{2}}}$$

where σ_1_ and σ_2_ are the estimates of standard deviations of *P*_1_ and *P*_2_, respectively, and *n*_1_ and *n*_2_ are the respective sample sizes of *Y*_1_ and *Y*_2_. With large sample sizes and the fact that *P*_1_ and *P*_2_ are known, the sampling distribution of *Ȳ*_1_ − *Ȳ*_2_ could be estimated through a normal distribution centered at μ_1_ − μ_2_ with the standard error given by equation 2. Instead, to avoid introducing extra variability from estimating σ_1_ and σ_2_ using sample variances *s*_1_ and *s*_2_, and to be able to handle the data with different sample sizes, Student’s *t* test can be used. Student’s *t* test assumes that samples *Y*_1_ and *Y*_2_ are both drawn from variables that follow a normal distribution and have equal variances, which can be estimated by a pooled variance combining the sample variances *s*_1_ and *s*_2_. Applying Student’s *t* test for samples with unequal variances or sample sizes can lead to unreliable conclusions with large type I and type II error probabilities (48).

### (iii) Welch's *t* test of the equivalence hypothesis.

Welch's *t* test can handle unequal variances or sample sizes in the data from the two sampling or sample processing approaches (30). The *t* statistics for Welch's *t* test of the hypothesis defined in equation 1 are

$$t_{1}=\frac{{\overset{¯}{Y}}_{1}-{\overset{¯}{Y}}_{2}-\Delta }{\sqrt{\frac{s_{1}^{2}}{n_{1}}+\frac{s_{2}^{2}}{n_{2}}}},t_{2}=\frac{{\overset{¯}{Y}}_{1}-{\overset{¯}{Y}}_{2}+\Delta }{\sqrt{\frac{s_{1}^{2}}{n_{1}}+\frac{s_{2}^{2}}{n_{2}}}}\;$$

and *H*_0_ is rejected if *t*_1_ is less than −*t*_α,df_ and *t*_2_ is greater than *t*_α,df_, where df is degrees of freedom.

However, the censored nature of the MIC data results in the presence of aggregated observations in the regions MIC ≤ *L* and MIC > *U*, where *L* and *U* are the smallest and largest drug concentrations tested, respectively. A long-tail in the left-hand end, MIC ≤ *L*, and a long-tail in the right-hand end, MIC > *U*, of the distribution are common (7). Such long-tailed shapes are common in the distributions even after the log_2_(MIC) transformation (12).

### (iv) Robust *t* test of the equivalence hypothesis.

As noted above, the log_2_(MIC) distributions most often do not follow a normal distribution. To improve robustness of the equivalence hypothesis testing and handle the long tails in the log_2_(MIC) distributions, we built upon Welch's *t* test to use the trimmed data and Winsorized variance. This is known as the robust *t* test (31). Let *Y*_1_ and *Y*_2_ be the ordered sample data, e.g., $Y_{1}=[y_{1,1},\;y_{1,2,}\;\cdots \;;\;{y_{1,n}}_{1}]$; then under *H*_0_:

$$t_{t_{1}}=\frac{{\overset{¯}{Y}}_{1,tg}-{\overset{¯}{Y}}_{2,tg}-\Delta }{\sqrt{\frac{s_{1,wg}^{2}}{n_{1(n_{1}-1)}}+\frac{s_{2,wg}^{2}}{n_{2}(n_{2}-1)}}}$$

$$t_{t_{2}}=\frac{{\overset{¯}{Y}}_{1,tg}-{\overset{¯}{Y}}_{2,tg}-\Delta }{\sqrt{\frac{s_{1,wg}^{2}}{n_{1(n_{1}-1)}}+\frac{s_{2,wg}^{2}}{n_{2}(n_{2}-1)}}}$$

where *Ȳ*_1,*tg*_ and *Ȳ*_2,*tg*_ are the trimmed (indicated by *t*) means and *g* is the number of the trimmed data points from each of the sides of the ordered *Y*_1_ and *Y*_2_ defined as

$${\overset{¯}{Y}}_{1,tg}=\frac{1}{n-2g}(y_{1,g+1}+y_{1,g+1}+\cdots \;+y_{1,g+1})$$

$${\overset{¯}{Y}}_{2,tg}=\frac{1}{n-2g}(y_{2,g+1}+y_{2,g+1}+\cdots \;+y_{2,g+1})$$

and the Winsorized (indicated by *w*) sum of squares $s_{1}^{2},wg$ and $s_{2}^{2},wg$ are defined as

$$s_{1}^{2},wg=\frac{1}{n_{1}}\left\{(g+1)y_{1,g+1}+y_{1,g+2}+\cdots +y_{1,n-g-1}+(g+1)y_{1,n_{1}-g}\right\}$$

$$s_{2}^{2},wg=\frac{1}{n_{2}}\left\{(g+1)y_{2,g+1}+y_{2,g+2}+\cdots +y_{2,n-g-1}+(g+1)y_{2,n_{2}-g}\right\}$$

The trimmed *t* statistics $t_{t_{1}}$ and $t_{t_{2}}$ each follow a *t* distribution with the degrees of freedom df (49)

$$\frac{1}{\text{df}}=\frac{c^{2}}{h_{1}-1}+\frac{{\left(1-c\right)}^{2}}{h_{2}-1}$$

where *c* is given by

$$c=\frac{\frac{s_{1}^{2}wg}{h_{1}\left(h_{1}-1\right)}}{\frac{s_{1}^{2}wg}{h_{1}\left(h_{1}-1\right)}+\frac{s_{2}^{2}wg}{h_{2}\left(h_{2}-1\right)}}$$

Thus, *H*_0_ is rejected if $t_{t_{1}}<-t_{\alpha ,\text{df}}\;\text{and}\;t_{t_{2}}>t_{\alpha ,\text{df}}$.

### (v) Power analysis of the statistical equivalence testing.

The power of testing statistical equivalence of the average log_2_(MIC) estimates for each antimicrobial drug from the two sampling or sample processing approaches for the target bacterial species in the stratum can be computed (using De Morgan's law [50]) as follows:

(3) $$\text{Power}=1-\text{P}(-t_{t_{1}}\leq t_{\alpha ,\text{df}}|H_{a})-\text{P}\left(t_{t_{2}}\leq t_{\alpha ,\text{df}}|H_{a}\right)$$

To compute the right-hand side of equation 3, we use the noncentral *t* distribution at $t_{t_{1}}$ and its cumulative distribution $\tau_{\text{df}}(0|t_{t_{1}})$ and a similar distribution of $t_{t_{2}}$:

$$\text{Power}=1-\tau_{\text{df}}(t_{\alpha ,\text{df}}|-t_{t_{1}})-\tau_{\text{df}}(t_{\alpha ,\text{df}}|-t_{t_{2}})$$

The test can be considered to have an acceptable power if the power is ≥0.80, following published guidelines (51, 52).

### Determining Δ_min_ from 95% confidence interval of the difference of the means.

The threshold difference Δ_min_ is the minimum Δ in equation 1 at which the two sampling or sample processing approaches still yield statistically nonequivalent estimates of susceptibility to the antimicrobial drug of the bacterial species in the sampled stratum. This threshold value for each antimicrobial and statistical test (Welch's *t* test or robust *t* test) was found from the data via a sequential algorithm repeating the test over a range of the difference values, starting from a large value [e.g., a 4-log difference between the average log_2_(MIC) estimates] and then shrinking Δ by a small step (0.01 log_2_) and retesting the *H*_0_ of a statistically significant difference between the average log_2_(MIC) estimates from the two approaches, until reaching the Δ_min_ value below which the *H*_0_ could no longer be rejected.

### Microbiological procedures. (i) Case studies 1 and 2.

Microbiological procedures used by the NARMS are described in the program's *Manual of Laboratory Methods* (53). Phenotypic susceptibility of the S. enterica isolates to antimicrobials is determined in the broth microdilution assay using the Sensititre system (TREK Diagnostic Systems Inc., Cleveland, OH), in accordance with the manufacturer recommendations and the Clinical and Laboratory Standards Institute (CLSI) guidelines (47, 53). The assays for the S. enterica isolates from cattle processing plants in 2013-2014 were performed using the Sensititre plate format CMV3AGNF as of those years, which included antimicrobials listed in Table 1. The assays for the nontyphoidal S. enterica isolates from humans in 2015 were performed using the Sensititre CMV3AGNF plate format as of that year and an additional plate format containing broad-spectrum β-lactams; the tested antimicrobials are listed in Table 2.

### (ii) Case study 3: sampling.

Fresh fecal pads (*n* = 32) were collected (the entire pad was lifted from the ground without mixing, placed into a sterile plastic bag, and transported while being kept horizontal) from different beef and dairy cattle at research facilities at Kansas State University during May to June 2016. The animals that were sampled had not received antimicrobial drugs in the preceding week but 1 to 3 weeks prior had received either antimicrobials (macrolides or tetracyclines to treat limited bovine respiratory disease or as a part of a research study) or feed supplemented with copper or zinc (these feed additives can coselect AMR in the animal fecal bacteria [11, 26–29]). This animal selection was done to ensure that the cattle fecal E. coli would have detectable but not uniformly high levels of AMR, to facilitate statistical analyses of the data. A collected fecal pad weighed 1.2 kg on average (5th and 95th percentiles: 0.4, 2.0). The average (5th, 95th percentile) pad dimensions were a length of 24 (18, 31) cm, a width of 18 (12, 25) cm, and a height of 3 (5, 9) cm.

### (iii) Sample processing.

On each fecal pad, four locations along the longest axis of the pad—its length—were marked using a sterile plastic loop. The locations were spread along the pad length (depending on the length) equidistantly ∼3 to 5 cm (∼1.5 to 2 in.) apart. Feces at the four locations were opened to the depth of ∼1 cm using sterile tools (to avoid the possibility of culturing E. coli that may have accidentally contaminated the pad exterior). One fecal aliquot of ∼1 g was aseptically collected from the bottom of the opening at each of the four locations. The locations were counted left to right. A random number was generated from a Uniform (1,4). When the aliquot from the location with the number corresponding to the generated random number was plated on a MacConkey agar plate, four E. coli colonies were obtained from the plate for isolation. When each of the aliquots from the other three locations on the pad was plated on a MacConkey agar plate, one E. coli isolate was obtained from the plate for isolation.

### (iv) Microbiological procedures.

Each fecal aliquot of ∼1 g was diluted in 10 ml of buffered peptone water (PBS) and vortexed gently until fully mixed. Of the supernatant, 100 μl was diluted 1:10 in sterile PBS and 100 μl was diluted 1:100 in sterile PBS. Of each of the dilutions, 100 μl was plated on a MacConkey agar plate and incubated at 37.5°C for 24 h. For the randomly selected aliquot on the pad, from the MacConkey plate with well-separated colonies, 4 typical coliform colonies chosen from different parts of the plate (convenience randomization) were each streaked on a tryptic soy broth supplemented with 5% sheep blood agar plate (BAP) and incubated at 37.5°C for 24 h. For each of the other three aliquots from the pad, from the MacConkey plate with well-separated colonies, one typical coliform colony (chosen via convenience randomization) was streaked on a BAP plate and incubated at 37.5°C for 24 h. Presumptive E. coli colonies from each BAP plate were subjected to the indole test, and the indole-producing ones were identified as E. coli. When needed, additional coliform colonies from the MacConkey plate were replated for isolation and subjected to the indole test to obtain the sought number of E. coli isolates from the fecal aliquot (i.e., one or four isolates). Phenotypic susceptibility to antimicrobials of each E. coli isolate was determined in the broth microdilution assay following the Sensititre plate manufacturer instructions and in accordance with the CLSI recommendations (47, 53). The Sensititre plate formats GN4F and BOPO6F as of 2016 were used. The strain E. coli ATCC 25922 was used for the quality control of the assays, along with the positive- and negative-control wells. The assay results on the plates were read on the Sensititre ARIS automated reading instrument (TREK Diagnostic Systems Inc., Cleveland, OH).

### Software.

The NARMS 2013 to 2015 data (publicly available) and the data for the fecal pads were gathered in Microsoft Office Excel (Microsoft, Inc., Redmond, WA). The data were imported into R 3.4, in which the statistical analyses were performed. All the figures were made in Python 3 (Python Software Foundation).
